# Direct Observation of Size-Dependent Phase Transition in Methylammonium Lead Bromide Perovskite Microcrystals and Nanocrystals

**DOI:** 10.1021/acsomega.2c04503

**Published:** 2022-10-26

**Authors:** Yanmei He, Kaibo Zheng, Paul F. Henry, Tönu Pullerits, Junsheng Chen

**Affiliations:** † Department of Chemical Physics and NanoLund, Lund University, P.O. Box 124, 22100 Lund, Sweden; ‡ Nano-Science Center & Department of Chemistry, University of Copenhagen, Universitetsparken 5, Copenhagen 2100, Denmark; § Department of Chemistry, Technical University of Denmark, DK-2800 Kongens Lyngby, Denmark; ∥ ISIS Pulsed Neutron Muon Facility, Rutherford Appleton Laboratory, Harwell Campus, Didcot OX11 0QX, United Kingdom

## Abstract

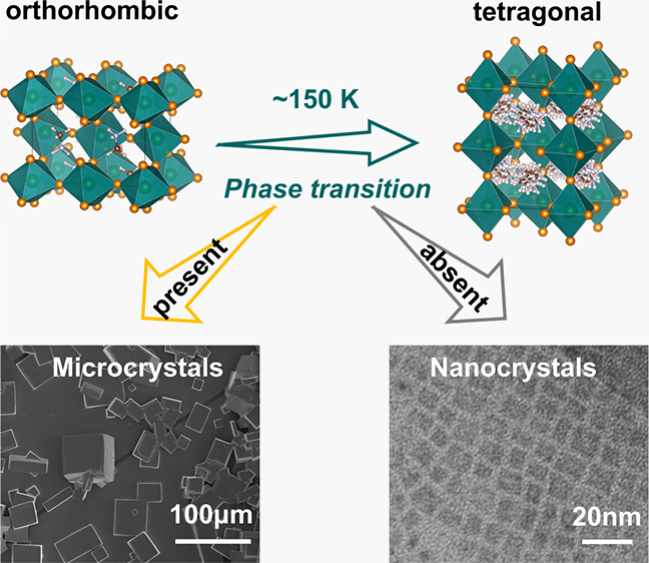

Methylammonium (MA) lead halide perovskites have been widely studied as active materials for advanced optoelectronics. As crystalline semiconductor materials, their properties are strongly affected by their crystal structure. Depending on their applications, the size of MA lead halide perovskite crystals varies by several orders of magnitude. The particle size can lead to different structural phase transitions and optoelectronic properties. Herein, we investigate the size effect for phase transition of MA lead bromide (MAPbBr_3_) by comparing the temperature-dependent neutron powder diffraction patterns of microcrystals and nanocrystals. The orthorhombic-to-tetragonal phase transition occurs in MAPbBr_3_ microcrystals within the temperature range from 100 to 310 K. However, the phase transition is absent in nanocrystals in this temperature range. In this work, we offer a persuasive and direct evidence of the relationship between the particle size and the phase transition in perovskite crystals.

## Introduction

Lead halide perovskites (LHPs) with the general formula APbX_3_ (X = Cl, Br, and I), where A is a cation such as cesium, methylammonium (MA), or formamidinium, show high performance as active materials for optoelectronic devices such as solar cells,^1^ light-emitting diodes (LEDs),^2,3^ displays (e.g., TVs),^3,4^ photodetectors,^5^ and lasers.^6^ The high performance of LHPs is related to their remarkable optical and electronic properties including broadband absorption, easily tunable optical band gap, efficient charge generation and transportation, and their bright and narrowband luminescence. These remarkable properties are closely related to their organic–inorganic hybrid structure, in which the lead-halide octahedra encapsulate organic cations in the unit cell. However, we are still lacking a thorough understanding about the relationship between the structure and efficiency of optoelectronic devices. As a result, significant challenges remain for accelerating the pace of the commercial applications of LHPs.

Optical and electrical properties of LHPs can be significantly altered by structural phase transitions^7−11^ and crystal size.^12−14^ Most LHPs undergo orthorhombic, tetragonal, and cubic phase transitions as the temperature increases.^8,10,15−19^ While the phase transition temperatures are dependent on their crystal size, for example, with reduced particle size, the phase transition might happen at lower temperatures. To investigate the size-dependent phase transition behavior, temperature-dependent steady-state photoluminescence (PL) and charge carrier mobility measurements have been used.^20−24^ By observing the abrupt peak shifts in the temperature-dependent PL spectra or the abrupt change of charge carrier mobility, the phase transition would be confirmed. However, these indirect techniques are not usually reliable and convenient to prove the existence of phase transitions in the bulk and nanocrystals because thermal expansion will also induce the emission peak shifts within a certain wavelength scale or mobility change in a certain range.

Temperature-dependent X-ray-based measurement is a direct method to monitor phase changes. However, in LHPs, the configuration of organic cations plays an important role in the temperature-dependent structural phase transitions; X-ray-based methods fail to provide direct information of the tumbling dynamics of the organic cation because scattering is dominated by the heavy elements. Neutron scattering offers a unique advantage to explore the phase transition behavior in LHPs because the neutron scattering lengths of the heavy (Pb) and light (C, H, N, etc.) elements are similar in magnitude. The differences in scattering lengths directly reveal any changes of organic cation orientation as well as the occurrence of phase transition,^25^ unachievable from XRD techniques.

In this work, we investigate the phase transition behaviors in MAPbBr_3_ microcrystals and nanocrystals with the aid of the temperature-dependent neutron powder diffraction. The measuring temperature changes from 100 to 310 K using a wide momentum transfer. Evaluation of Bragg peak changes in the powder diffraction patterns allows direct observation of structural phase transition differences in nanocrystals and microcrystals within this temperature range. Furthermore, we carefully discuss the possible mechanisms of size-dependent phase transition.

## Results and Discussion

**Figure 1:**
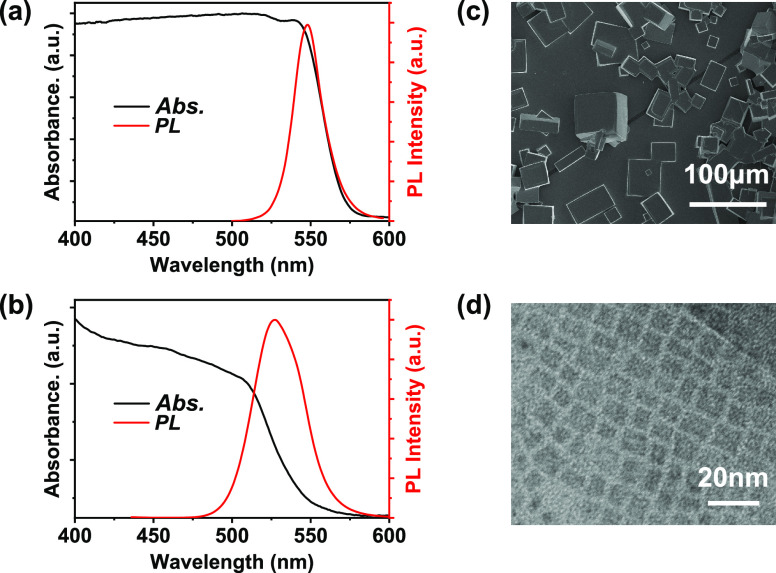
Absorption (black line) and PL (red line) of MAPbBr_3_ microcrystals (a) and nanocrystals (b). SEM image of MAPbBr_3_ microcrystals (scale bar = 100 μm) (c) and TEM image of MAPbBr_3_ nanocrystals (scale bar = 20 nm) (d).

MAPbBr_3_ microcrystals were synthesized by a modified solution inverse temperature crystallization method (see the Supporting Information (SI) for details). The scanning electron microscope (SEM) image shows the microcrystals with length scales of a few tens of micrometers (Figure 1) and with a thickness of about 2–5 μm. The absorption and PL spectra of the MAPbBr_3_ microcrystals are shown in Figure 1. The absorption intensity of MAPbBr_3_ microcrystals shows a very weak increase above the band gap. This phenomenon is ascribed to the partial coverage of the microcrystals on the substrate during the measurement.^26^ MAPbBr_3_ nanocrystals were prepared by using a ligand-assisted reprecipitation method^27^ (see the Supporting Information (SI) for details). The transmission electron microscope (TEM) image shows the nanocrystals with a size of about 7 nm (see Figure S1). The absorption and PL spectra of the MAPbBr_3_ nanocrystals are shown in Figure 1. Due to the quantum confinement effect,^28^ the PL spectrum of MAPbBr_3_ nanocrystals is blue shifted compared with that of MAPbBr_3_ microcrystals. The Gaussian peak fitting analysis is used in the PL spectra of both nanocrystals and microcrystals (Figures S2 and S3 and Table S1). From these results, there exist two peaks in both crystals. The fitting peak at 556 nm in nanocrystals and 566 nm in microcrystals can be ascribed to the defect emissions.^29−31^ The intrinsic PL full width at half-maximum (FWHM) of nanocrystals (at 528 nm) is around 34 nm, which is broader than that of microcrystals (at 547 nm, FWHM ∼21 nm). This indicates that the larger internal strain energy might exist in nanocrystals.^24,32,33^

**Figure 2:**
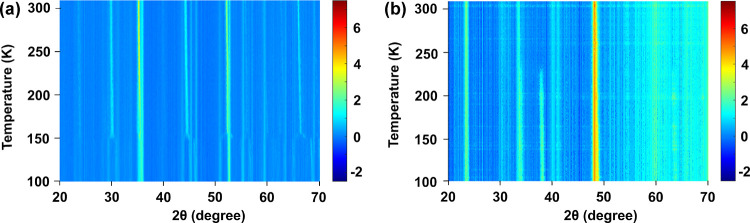
Contour plots of temperature-dependent neutron powder scattering patterns measured from MAPbBr_3_ microcrystals (a) and nanocrystals (b) within the temperature range from 100 to 310 K.

Neutron powder diffraction patterns of MAPbBr_3_ microcrystals and nanocrystals were measured on the thermal neutron diffractometer D20 at the Institut Laue–Langevin (ILL).^34^ A neutron wavelength of 1.87 Å (Ge(511)) was used to obtain a complete powder scattering pattern over a 2θ range of 153.6°. To study the temperature-dependent phase transition, the neutron powder scattering patterns were recorded in the temperature range from 100 to 310 K.^35^ For interpretation, we focus on the scattering angles between 20 and 70° (Figure 2), and unit cell planes are defined by the Miller index (*hkl*) and labeled in Figures S4–S6. In this scattering angle range, the microcrystals show considerable differences of scattering peaks in the whole temperature range (Figure 2a and Figures S4–S6). At a low temperature (<150 K), the MAPbBr_3_ microcrystals’ principal Bragg reflections are located at the angles of 31, 32.8, 37.2, 44.8, 46.4, 47.4, 51.4, 53.4, 55.8, 57, 57.5, 59.4, 63.8, 65, and 68.8^°^. Meanwhile, at 150 K, we observe abrupt changes in the scattering pattern, the well-known orthorhombic-to-tetragonal phase transition.^21,22,36^ Above 150 K, the main Bragg peaks are located at the angles of 31.6, 37, 45.5, 52.8, 56, 60.3, and 66.5^°^. Among these, the (221), (322), and (531) are representative of the tetragonal phase (Figures S4–S6). However, as shown in Figure 2b, MAPbBr_3_ nanocrystals present the unchanged principal Bragg reflections within the measured temperature range, indicating the absence of any phase transformation. Here, we find that the scattering peak at 38° disappears at a temperature above 225 K in MAPbBr_3_ nanocrystals. It might be ascribed to the residual of the nanocrystals’ surface capping agents: oleic acid. Oleic acid exhibits a crystalline structure (α or γ phase) at a low temperature and melts into an amorphous state, resulting in the disappearance of this scattering peak.^37,38^

**Figure 3:**
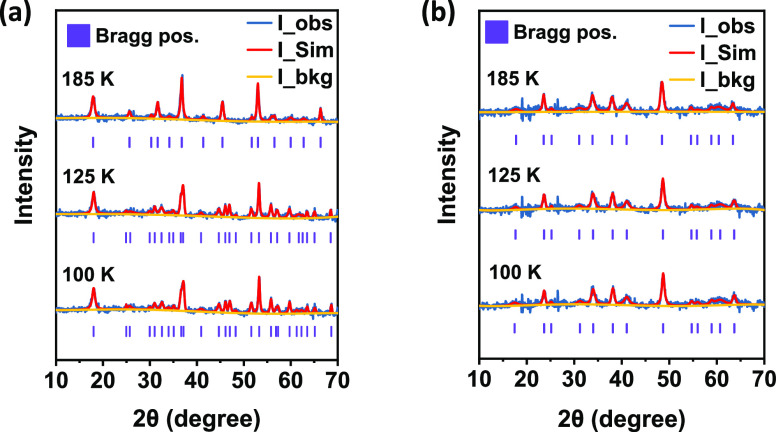
Rietveld refinement profiles of MAPbBr_3_ microcrystals (a) and nanocrystals (b) at different temperatures. The red line represents the calculated data, comparing with the experimental data (cyan line). The yellow lines represent the fitting background data. The vertical purple bars represent the simulated Bragg reflection positions.

**Figure 4:**
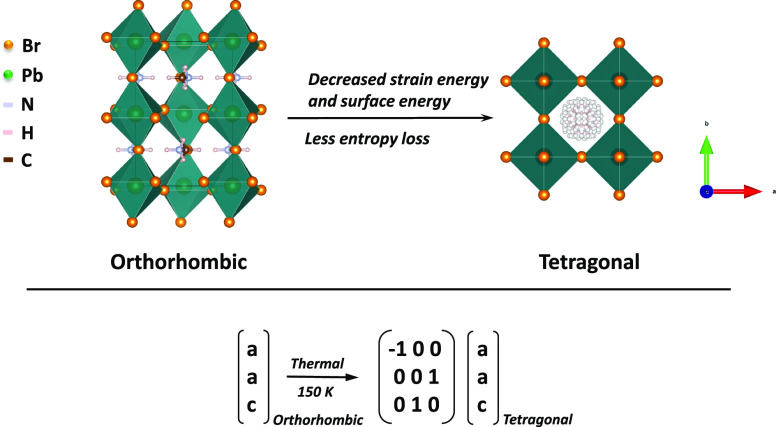
Schematic polyhedral plotting shows the phase transition mechanism in MAPbBr_3_ microcrystals. Below transformation matrices map the *c* axis from orthorhombic to tetragonal around transition temperature.

To further investigate the size-dependent phase transition, we carried out Rietveld refinement at different temperatures. As shown in Figure 3a, for MAPbBr_3_ microcrystals, the calculated intensities fit well with the experimental data at 100, 125, and 185 K. The simulated Bragg positions of MAPbBr_3_ microcrystals characterized at 100 and 125 K are consistent with the theoretical scattering peaks. When the temperature increases to over 150 K, the phase transition occurs (Figure 4). The number of Bragg scattering peaks reduces together with obvious peak shifting (to smaller scattering angles). To visualize the changes of peak location, we also plotted the neutron scattering patterns of MAPbBr_3_ microcrystals and nanocrystals at different scattering angle ranges, which were measured at 100 and 185 K (Figures S4–S6). The neutron scattering patterns of the microcrystals show the expected phase changes as reported.^21^ Meanwhile, we do not observe any obvious changes of Bragg positions or peak shifts in MAPbBr_3_ nanocrystals (Figure 3b and Figures S4–S6). It means that the nanocrystals show no phase changes (i.e., no orthorhombic-to-tetragonal phase transition).

The known phase transition behavior in bulk/microcrystalline MAPbBr_3_ is suppressed by reducing the particle size to nanometer. The different temperature-dependent phase transition behaviors in MAPbBr_3_ bulk crystals and nanocrystals can impact their optical and electrical properties and their corresponding applications. For example, when the temperature increases (from 77 to 298 K), at 150 K, a sudden red shift of the emission peak and reduction of exciton lifetime are observed in MAPbBr_3_ microcrystals. However, such spectral and exciton lifetime changes are absent in MAPbBr_3_ nanocrystals.^39^ Duan and coworkers^24^ reported that in MAPbBr_3_ microplates, the charge transporting ability became weaker with increasing temperature from 77 to 296 K. The temperature-dependent electron mobility shows an abrupt jump at around phase transition temperature points (130–170 K). The abrupt jump of the electron mobility shows a thickness dependence: the thinner the microplates are, the lower the abrupt jump temperature. This can play an important role in the electronic application of MAPbBr_3_ microplates at different temperatures.^24^ Based on these results, our results showed a constructive guidance to well understand the important role of size-dependent phase transition.

Different phase transition behaviors between the bulk and nanoparticles have been reported in different materials, such as BaTiO_3_, PbTiO_3_, SrBi_2_Ta_2_O_9_, and Bi_4_Ti_3_O_12_.^23,40−44^ The structural phase transition temperature of nanoparticles has been reported to be lower than their bulk counterparts. To explain this, several mechanisms have been proposed, such as the lack of nucleation sites in nanoparticles,^45,46^ increased internal pressure/strain in nanoparticles,^47,48^ and surface energy difference between nanomaterials and bulk materials.^32^ In our work, the internal strain in nanocrystals might be higher than that of microcrystals, as indicated by the broadened PL spectrum (Figure 1), which may play a role in the absence of phase transition compared with microcrystals. On the other hand, the MAPbBr_3_ nanocrystals have a larger surface-to-volume ratio than microcrystals, leading to the increased surface energy per unit volume compared to that of microcrystals.^42^ Meanwhile, in nanocrystals, the free rotation/tumbling of the organic cation MA is no longer restricted by the long-range dipole–dipole interaction between the polar MA cations. By releasing the long-range order interaction, the MA cations will gain a higher degree of freedom in nanocrystals. As a result, the vibrational and configurational entropy losses are introduced, which could cause different phase transition behaviors as well.^22,23^ In short, all the above-mentioned factors could contribute to the absence of structural transformation in MAPbBr_3_ nanocrystals within the experimental temperature range from 100 to 310 K. It is out of the scope of this work to discuss which factor has the dominant contribution. To address this critical issue, further experimental and theoretical works would be needed.

## Conclusions

In summary, we measured neutron powder diffraction patterns of MAPbBr_3_ microcrystals and nanocrystals. From the experimental results, we observed different phase transition behaviors: the orthorhombic-to-tetragonal phase transition happens at around 150 K in microcrystals, but it is absent in nanocrystals. Different factors may cause the absence of such phase transition in MAPbBr_3_ nanocrystals between 100 and 310 K, including the increased strain energy and surface energy, as well as the introduced entropy loss because of the released long-range cations’ dipole–dipole interaction. Our results directly show the different temperature-dependent phase transition behaviors in MAPbBr_3_ microcrystals and nanocrystals, which is helpful to explain their different temperature-dependent optical and electrical properties. The size-dependent phase transition behavior may also exist in other types of halide perovskite materials. Hence, our finding will shed light on fabricating halide perovskite-based optoelectronic devices that operate at not only room temperature but also a low-temperature environment such as in the space.

## Supporting Information Available

Materials; preparation and characterization of MAPbBr_3_ microcrystals and nanocrystals; the size distribution of MAPbBr_3_ nanocrystals; and the neutron scattering spectra of MAPbBr_3_ microcrystals and nanocrystals at different angles and temperatures (PDF)
