# Supplementary material for: Direct Observation of Size-Dependent Phase Transition in Methylammonium Lead Bromide Perovskite Microcrystals and Nanocrystals
Source: ACS Omega. 2022 Oct 26;7(44):39970–4. doi: 10.1021/acsomega.2c04503 (PMC9648073; doi:10.1021/acsomega.2c04503)
Supplement: Supplementary file 1 — ao2c04503_si_001.pdf [file ao2c04503_si_001.pdf]

Supporting Information

Direct Observing Size-Dependent Phase Transition in Methylammonium Lead Bromide Perovskite Microcrystals and Nanocrystals

Yanmei He, [1] *Kaibo Zheng,* [1,3] *Paul F. Henry,* [4] *Tönu pullerits,* [1] *Junsheng Chen,* [*,1,2]

[1] Department of Chemical Physics and NanoLund, Lund University, P.O. Box 124, 22100 Lund, Sweden

[2] Nano-Science Center & Department of Chemistry, University of Copenhagen, Universitetsparken 5, 2100 Copenhagen, Denmark

[3] Department of Chemistry, Technical University of Denmark, DK-2800 Kongens Lyngby, Denmark

[4] ISIS Pulsed Neutron Muon Facility, Rutherford Appleton Laboratory, Harwell Campus, Didcot OX11 0QX, United Kingdom

AUTHOR INFORMATION

Corresponding Author

*E-mail: junsheng.chen@chemphys.lu.se

S1. Materials

Lead bromide ($PbBr_2$ 98%), oleylamine (OAm, 80−90%) and oleic acid (OA, 90%) were purchased from Sigma Aldrich. Methylammonium bromide (MABr, CH_3NH_3Br, 98%) was purchased from Dyenamo. All compounds were used as received without any further purification.

S2. $MAPbBr_3$ microcrystals and nanocrystals preparation and characterization

To synthesize $MAPbBr_3$ microcrystals, a precursor solution was prepared by dissolving 1.24 g of MABr and 3.67 g of $PbBr_2$ in 10 mL dry dimethylformamide (DMF) at room temperature. The precursor solution was then put on a hot plate (110 ℃) to grow the $MAPbBr_3$ single crystals. The $MAPbBr_3$ microcrystals were precipitated within 5 minutes (at 110 ℃) and collected. The collected microcrystals were ground into powder for neutron powder scattering measurement.

$MAPbBr_3$ nanocrystals were prepared by using ligand-assisted re-precipitation method reported by Zhang et al.[1] 0.0179 g (0.16 mmol) of MABr and 0.0734 g (0.2 mmol) $PbBr_2$ were dissolved in 5 mL dry DMF, then 50 µL dry OAm and 0.5 mL OA were added to form the $MAPbBr_3$ precursor solution. 250 µL as-prepared precursor solution was swiftly injected into pre-heated hot toluene solution (60 ℃ in an oil bath) under vigorous stirring. The reaction was kept for 5 minutes, then was stopped by cooling in an ice-water bath. The ice-water cooled crude was centrifuged at 4000 rpm for 5 minutes, and the supernatant was collected and centrifuged again at 6500 rpm for 10 minutes. After centrifugation, the supernatant was discarded, and the particles were re-dispersed in heptane. For neutron powder scattering measurement, we washed the surface capping agents of $MAPbBr_3$ nanocrystals. The as-prepared nanocrystals in heptane were mixed with acetone (Vheptane/Vacetone=1:3) and the nanocrystals were centrifuged at 6500 rpm for 10min. The washing procedure was repeated twice. The obtained nanocrystals without surface capping agents were dried under vacuum (10^{-3} mbar) for 3 hours. The dried nanocrystals were ground into powder for neutron powder scattering measurement.

S3. $MAPbBr_3$ microcrystals and nanocrystals characterization

The prepared $MAPbBr_3$ microcrystals and nanocrystals were characterized by steady-state UV-vis absorption and photoluminescence (PL) spectra and scanning electron microscopy (SEM) or transmission electron microscopy (TEM). The steady-state UV-vis absorption spectra were obtained using a PerkinElmer Lambda 1050 spectrophotometer. The steady-state fluorescence spectra were recorded using a standard spectrofluorometer (Spex1681) with excitation at 400 nm. The size of $MAPbBr_3$ nanocrystals was characterized by high-resolution analytical TEM (Jeol 3000F). The corresponding distribution histograms are shown in Figure S1. The mean sizes of $MAPbBr_3$ and nanocrystals were obtained by fitting the size distribution with a Gaussian function.

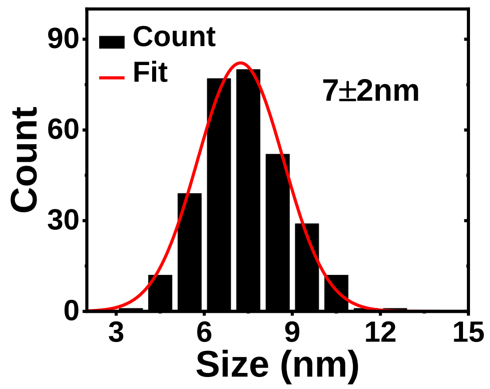

Figure S1. Size distribution of $MAPbBr_3$ NCs.

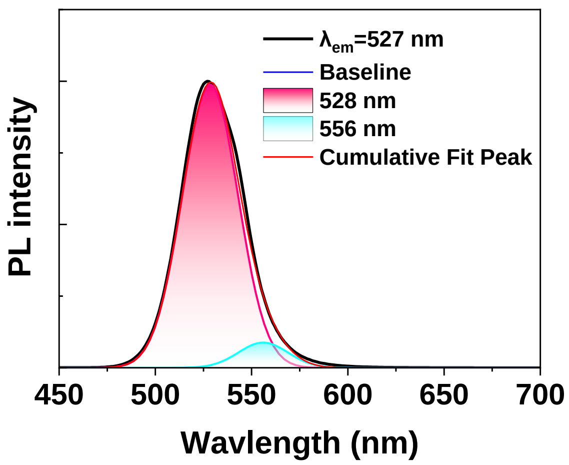

Figure S2. Gaussian Peak fitting of the PL spectrum of $MAPbBr_3$ nanocrystals.

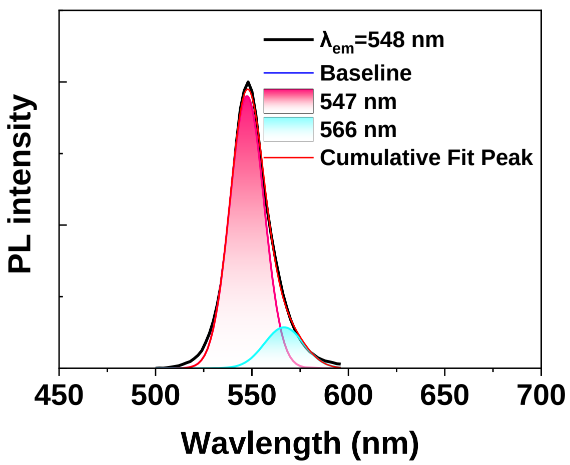

Figure S3. Gaussian Peak fitting of PL spectrum of $MAPbBr_3$ microcrystals.

Table S1. The gaussian peak fitting data of the PL spectra of $MAPbBr_3$ nanocrystals and microcrystals are summarized, including FWHM and peak position.

Materials	Fitting Peak 1	Fitting Peak 2
	FWHM/ Peak position	FWHM/ Peak position
Nanocrystal	34 nm/ 528 nm	30 nm/ 556 nm
Microcrystal	21 nm/ 547 nm	24 nm/ 566 nm

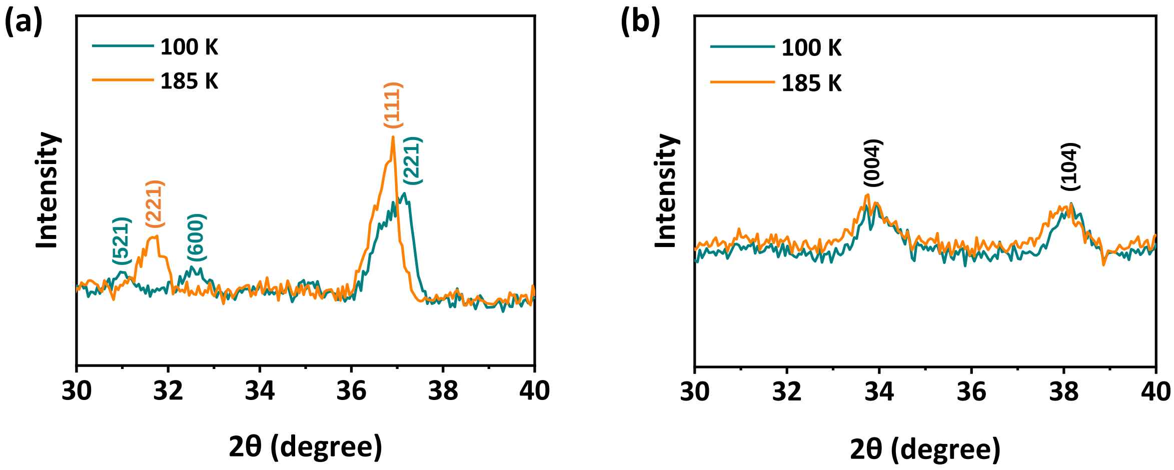

Figure S4. The neutron scattering spectra of $MAPbBr_3$ microcrystals (a) and nanocrystals (b) with the angles changing from 30 to 40° are shown here. The dark green and orange lines stand for the experimental data of 100 and 185 K, respectively.

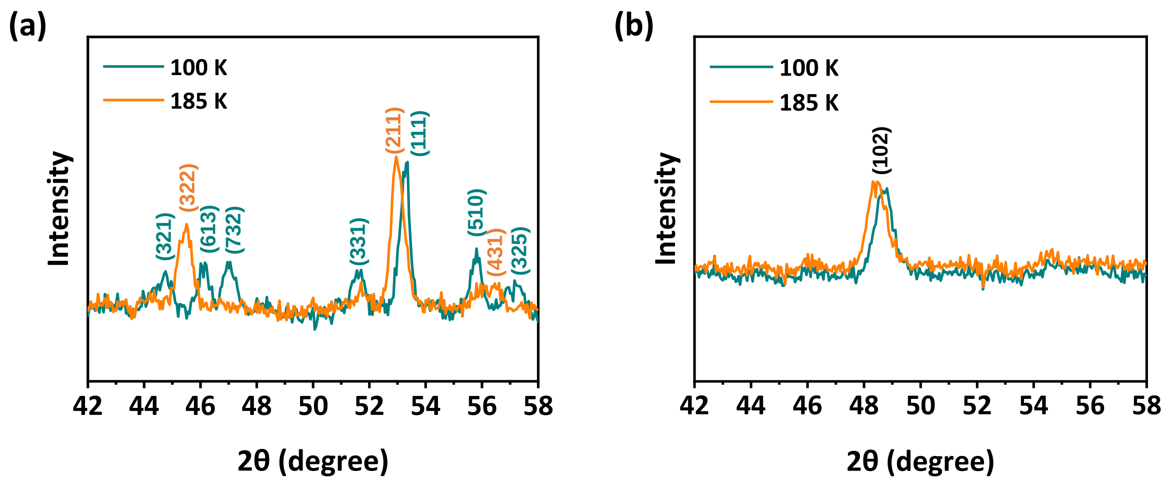

Figure S5. The neutron scattering spectra of $MAPbBr_3$ microcrystals (a) and nanocrystals (b) with the angles changing from 42 to 58° are shown here. The dark green and orange lines stand for the experimental data of 100 and 185 K, respectively.

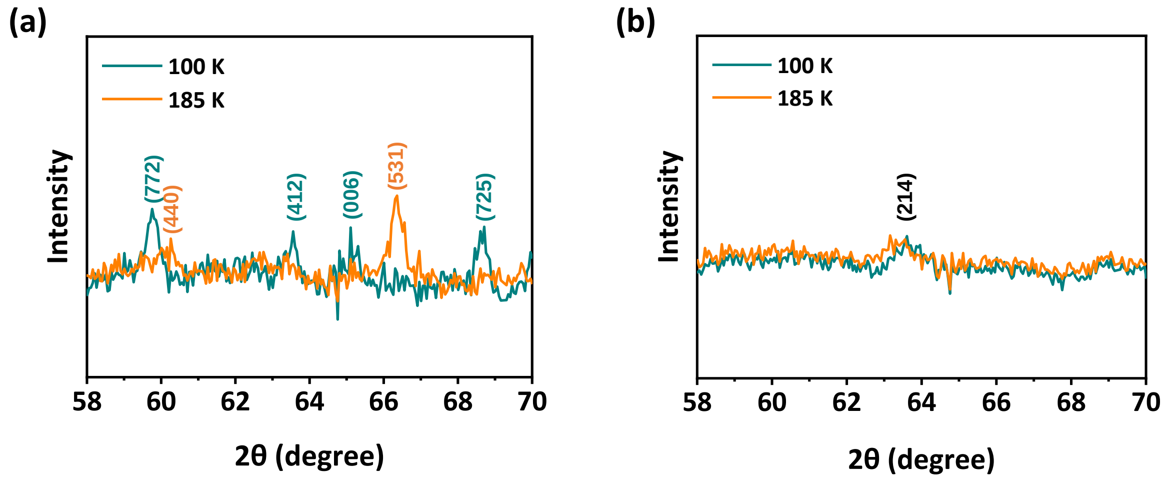

Figure S6. The neutron scattering spectra of $MAPbBr_3$ microcrystals (a) and nanocrystals (b) with the angles changing from 58 to 70° are shown here. The dark cyan and orange lines stand for the experimental data of 100 and 185 K, respectively.

References

1. Zhang, F.; Zhong, H. Z.; Chen, C.; Wu, X. G.; Hu, X. M.; Huang, H. L.; Han, J. B.; Zou, B. S.; Dong, Y. P. Brightly Luminescent and Color-Tunable Colloidal $CH_3NH_3PbX_3$ (X = Br, I, Cl) Quantum Dots: Potential Alternatives for Display Technology. *ACS Nano* **2015,** *9* (4), 4533-4542.
